# Peripheral blood transcriptomic sub-phenotypes of pediatric acute respiratory distress syndrome

**DOI:** 10.1186/s13054-020-03410-7

**Published:** 2020-12-07

**Authors:** Nadir Yehya, Brian M. Varisco, Neal J. Thomas, Hector R. Wong, Jason D. Christie, Rui Feng

**Affiliations:** 1 Department of Anesthesiology and Critical Care Medicine, 6040A Wood Building, Children’s Hospital of Philadelphia, 3401 Civic Center Boulevard, Philadelphia, PA 19104 USA; 2 University of Pennsylvania, Philadelphia, PA USA; 3 Division of Critical Care Medicine, Department of Pediatrics, Cincinnati Children’s Hospital Medical Center, Cincinnati, OH USA; 4 College of Medicine, University of Cincinnati, Cincinnati, OH USA; 5 Division of Pediatric Critical Care Medicine, Department of Pediatrics and Public Health Science, Penn State Hershey Children’s Hospital, Hershey, PA USA; 6 Critical Care Division, Department of Medicine, Pulmonary, Allergy, Perelman School of Medicine, University of Pennsylvania, Philadelphia, PA USA; 7 Center for Translational Lung Biology, Perelman School of Medicine, University of Pennsylvania, Philadelphia, PA USA; 8 Center for Clinical Epidemiology and Biostatistics, Perelman School of Medicine, University of Pennsylvania, Philadelphia, PA USA; 9 Department of Biostatistics, Center for Clinical Epidemiology and Biostatistics, Epidemiology, and Informatics, University of Pennsylvania, Philadelphia, PA USA

**Keywords:** Children, ARDS, PARDS, Endotypes, Gene expression, Sub-phenotypes

## Abstract

**Background:**

Acute respiratory distress syndrome (ARDS) is heterogeneous and may be amenable to sub-phenotyping to improve enrichment for trials. We aimed to identify subtypes of pediatric ARDS based on whole blood transcriptomics.

**Methods:**

This was a prospective observational study of children with ARDS at the Children’s Hospital of Philadelphia (CHOP) between January 2018 and June 2019. We collected blood within 24 h of ARDS onset, generated expression profiles, and performed k-means clustering to identify sub-phenotypes. We tested the association between sub-phenotypes and PICU mortality and ventilator-free days at 28 days using multivariable logistic and competing risk regression, respectively.

**Results:**

We enrolled 106 subjects, of whom 96 had usable samples. We identified three sub-phenotypes, dubbed CHOP ARDS Transcriptomic Subtypes (CATS) 1, 2, and 3. CATS-1 subjects (*n* = 31) demonstrated persistent hypoxemia, had ten subjects (32%) with immunocompromising conditions, and 32% mortality. CATS-2 subjects (*n* = 29) had more immunocompromising diagnoses (48%), rapidly resolving hypoxemia, and 24% mortality. CATS-3 subjects (*n* = 36) had the fewest comorbidities and also had rapidly resolving hypoxemia and 8% mortality. The CATS-3 subtype was associated with lower mortality (OR 0.18, 95% CI 0.04–0.86) and higher probability of extubation (subdistribution HR 2.39, 95% CI 1.32–4.32), relative to CATS-1 after adjustment for confounders.

**Conclusions:**

We identified three sub-phenotypes of pediatric ARDS using whole blood transcriptomics. The sub-phenotypes had divergent clinical characteristics and prognoses. Further studies should validate these findings and investigate mechanisms underlying differences between sub-phenotypes.

## Introduction

Acute respiratory distress syndrome (ARDS) is characterized by acute onset of bilateral pulmonary edema and hypoxemia not fully explained by cardiac dysfunction [1, 2]. Primarily defined for adults, ARDS affects 45,000 children in the USA annually [3], representing 10% of mechanically ventilated children in pediatric intensive care units (PICUs) [4], with a mortality rate of 20% in the USA and 30% worldwide [5, 6]. There are no specific pharmacological therapies for adult or pediatric ARDS despite several trials, and supportive care with lung-protective ventilation [7] and fluid restriction [8] remains the mainstay of treatment.

ARDS is heterogeneous, with patients having distinct comorbidities and inciting etiologies. This heterogeneity has contributed to negative trial results, as therapies effective in some patients are ineffective in others [9]. Methods to reduce heterogeneity, including sub-phenotyping using protein and mRNA biomarkers, have been proposed for improving patient selection for future clinical trials [10]. Extensive work in adult ARDS has demonstrated differential response to positive end-expiratory pressure [11], conservative fluid management [12], and simvastatin [13] depending on subtypes defined, in part, by protein biomarkers. By contrast, the presence of subtypes in pediatric ARDS is largely unexplored [14].

Whole blood transcriptomics has led to significant insights into the heterogeneity of adult [15, 16] and pediatric sepsis [17, 18]. Unsupervised clustering has identified sepsis subtypes with differential biology, and potentially differential response to therapy [19]. Few gene expression studies have been performed in adult ARDS [20], and none in pediatrics. The aim of the present study was to identify sub-phenotypes of pediatric ARDS using unsupervised clustering on whole blood transcriptomics, hypothesizing that ≥ 2 subtypes would be identified.

## Methods

### Study design and subjects

This was a prospective cohort study approved by the Children’s Hospital of Philadelphia’s (CHOP) Institutional Review Board between January 1, 2018, and June 30, 2019, with informed consent obtained prior to enrollment [21, 22]. Inclusion criteria were (1) acute (≤ 7 days of risk factor) respiratory failure requiring invasive mechanical ventilation, (2) arterial access, (3) age > 1 month and < 18 years, (4) Pao_2_/Fio_2_ ≤ 300 on two consecutive arterial blood gases separated by ≥ 1 h on positive end-expiratory pressure (PEEP) ≥ 5 cmh_2_o, and (5) bilateral infiltrates on radiograph. Exclusion criteria were (1) respiratory failure from cardiac failure (by echocardiography), (2) exacerbation of underlying chronic lung disease, (3) chronic ventilator dependence, (4) cyanotic heart disease, (5) ventilation for > 7 days before Pao_2_/Fio_2_ ≤ 300, (6) ARDS established outside of CHOP, (7) inability to obtain consent, or (8) prior enrollment.

### Procedures

Clinical data were recorded prospectively. Blood was collected ≤ 24 h of ARDS onset (time of fulfilling all Berlin criteria) in PAXgene RNA tubes (BD Biosciences, San Jose, CA), kept overnight at room temperature up to 24 h, and then stored at − 20 °C for batched analysis. After ensuring RNA integrity, we generated gene expression profiles using Human Gene 2.1 ST Array (Affymetrix, Santa Clara, CA) and the GeneTitan instrument. Microarray data were background-corrected and quantile-normalized using robust multi-array average for downstream analyses [23]. Data were uploaded to the Gene Expression Omnibus (GSE147902).

### Definitions

Oxygenation index equaled: (mean airway pressure × FIO_2_ × 100)/PaO_2_ (in mmHg). Vasopressor score [24] was: dopamine (µg/kg/min) × 1 + dobutamine (µg/kg/min) × 1 + epinephrine (µg/kg/min) × 100 + norepinephrine (µg/kg/min) × 100 + phenylephrine (µg/kg/min) × 100 + milrinone (µg/kg/min) × 10 + vasopressin (U/kg/min) × 10,000. Severity of illness score was the Pediatric Risk of Mortality (PRISM) III at 12 h. Nonpulmonary organ failures were defined using accepted definitions [25]. The designation of “immunocompromised” required presence of an immunocompromising diagnosis (oncologic, immunologic, rheumatologic, transplant) and active immunosuppressive therapy, or presence of a congenital immunodeficiency [26].

### Outcomes

The objective of this study was to identify sub-phenotypes of pediatric ARDS and assess the association of these subtypes with clinical variables, PICU mortality, and ventilator-free days (VFDs) at 28 days. Only invasive ventilation was counted, with the first day as ARDS onset. Liberation from invasive ventilation for > 24 h defined ventilator duration. Patients requiring re-intubation > 24 h after extubation had additional days counted toward total ventilator days. VFDs were determined by subtracting total ventilator days from 28 in survivors. Patients with total ventilator days ≥ 28 days and all PICU nonsurvivors were assigned VFD = 0.

### Statistical analysis

For sub-phenotype discovery, we analyzed gene expression using k-means clustering, restricting the analysis to 31,136 annotated genes (as of July 2019). We chose an optimal number of clusters *k* using the gap statistic and 95% confidence intervals (CI). First, we computed the gap statistic and 95% CI for *k* = 1–10, considering clusters with overlapping confidence intervals as having similar performance (Additional file 1: Fig. 1). We then chose the maximal gap statistic with > 10 subjects per cluster (~ 10% of entire cohort). Clustering was performed solely based on gene expression, blinded to clinical characteristics and outcomes. For pathway analysis of the identified sub-phenotypes, probes were filtered for expression values ≥ 10 in ≥ 10 samples and differentially expressed genes (DEGs) for each subtype determined using DESeq2 [27] with BioMaRT [28]. Twofold upregulated and downregulated DEGs were analyzed in Ingenuity Pathway Analysis (IPA) [29] and ToppGene [30] to identify predicted upstream regulators, Gene Ontogeny terms, and key pathways. Pathways with *q* value < 0.1 are presented in Supplement.

Sub-phenotypes were assessed for association with clinical characteristics using nonparametric statistics. Categorical data were compared using Fisher's exact test. We tested the association between sub-phenotypes and mortality and VFDs using logistic and competing risk regression [31], respectively, adjusting for (individually and together) immunocompromised status and PRISM III score. We reasoned that these two variables plausibly contributed to both the identity of the sub-phenotypes and outcomes, as they are associated with circulating immune cell gene expression and pulmonary and nonpulmonary severity of illness. Thus, immunocompromised status and PRISM III represent potential confounding of the association between subtypes and outcomes. Separately, we tested the association between sub-phenotypes and outcome adjusting for predicted mortality based on a recent pediatric ARDS-specific mortality prediction score [32]. Additionally, we repeated the above regressions while also adjusting for absolute neutrophil count (ANC) and absolute lymphocyte count (ALC) in order to assess whether associations between sub-phenotypes and outcomes were driven by lymphocyte subset proportions. Due to the limited number of deaths in the cohort, we restricted the number of confounders in all models to minimize bias and variance. Analyses were performed in Stata 14.2/SE (StataCorp, LP, College Station, TX) and R 3.0.1 (www.r-project.org). Heatmaps were generated with pheatmap and gridExtra in R.

## Results

**Fig. 1:**
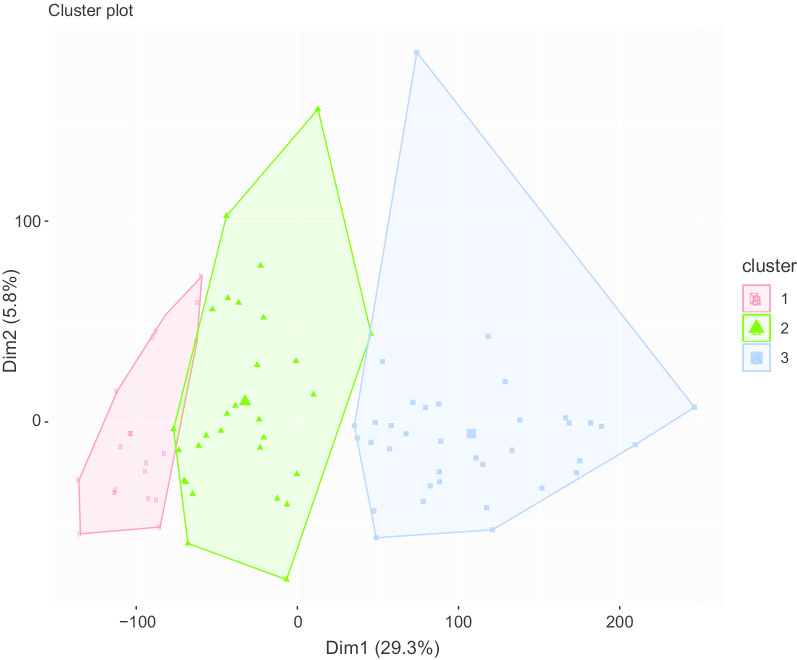
Three clusters identified using unsupervised k-means clustering, dubbed CHOP ARDS Transcriptomic Subtypes (CATS) 1 (red), 2 (green), and 3 (blue). The individual subjects are plotted in a two-dimensional plot, with the principle dimensions (Dim 1 and 2) which account for 29.3% and 5.8% of the variance, as the axes

Between January 2018 and June 2019, 140 children had ARDS. We consented and enrolled 106 subjects (76%), of whom 96 had usable samples (excluded eight for low RNA yield due to leukopenia and two for poor-quality RNA). Of these 96 subjects, 20 (21%) were nonsurvivors. Considering cluster gap statistic, 95% CI overlap and cluster size, *k* = 3 was chosen (Fig. 1). Sub-phenotypes were designated CHOP ARDS Transcriptomic Subtypes (CATS) 1, 2, and 3 and did not differ in severity of illness, ARDS etiology, or ARDS severity at onset (Table 1). Sub-phenotypes differed by proportion of immunocompromised subjects, with CATS-1 (32%) and CATS-2 (48%) having more immunocompromised subjects, relative to CATS-3 (14%; *p* = 0.011). CATS-1 had worse hypoxemia at 24 h, relative to the other subtypes.

**Table 1:** Demographics stratified by CHOP ARDS Transcriptomic Subtypes (CATS)

Variables	CATS-1 (*n* = 31)	CATS-2 (*n* = 29)	CATS-3 (*n* = 36)	*p* value
Age (years)	6.8 [1.2, 13]	14.1 [6.9, 16.5]	7 [1.8, 12.8]	0.006
Female (%)	13 (42)	12 (42)	13 (36)	0.898
Severity of illness				
PRISM III at 12 h	9 [5, 15]	13 [8, 18]	12 [7, 21]	0.278
Nonpulmonary organ failures	2 [1, 3]	1 [1, 2]	1 [1, 2]	0.530
Vasopressor score	10 [4, 18]	5 [0, 12]	7 [0, 28]	0.313
Comorbidities (%)				
Immunocompromised	10 (32)	14 (48)	5 (14)	0.011
Stem cell transplant	4 (13)	8 (28)	2 (6)	0.040
Cause of ARDS (%)				
Direct	22 (71)	23 (79)	25 (69)	0.652
Indirect	9 (29)	6 (21)	11 (31)	
Cause of ARDS (%)				
Infectious	21 (68)	25 (86)	29 (81)	0.211
Noninfectious	10 (32)	4 (14)	7 (19)	
Cause of ARDS (%)				
Infectious pneumonia	16 (52)	19 (66)	21 (58)	
Nonpulmonary sepsis	5 (16)	6 (21)	8 (22)	
Aspiration pneumonia	4 (13)	3 (10)	3 (8)	0.606
Trauma	1 (3)	1 (3)	1 (3)	
Other	5 (16)	0	3 (8)	
ARDS onset				
PaO_2_/FIO_2_	152 [88, 243]	148 [121, 222]	138 [85, 187]	0.450
OI	11.1 [6.7, 18.1]	11 [8.7, 13.4]	13.5 [9.6, 23.9]	0.371
24 h after onset				
PaO_2_/FIO_2_	179 [130, 240]	236 [182, 292]	252 [186, 323]	0.002
OI	9.8 [6.2, 14.3]	5.7 [4.8, 8.1]	6 [4.3, 10.6]	0.004
Outcomes				
Ventilator days (all)	7 [5, 12]	10 [5, 24]	6 [4, 10]	0.152
Ventilator days (survivors)	7 [6, 12]	9 [5, 16]	6 [4, 10]	0.201
VFDs at 28 days	16 [0, 22]	16 [0, 21]	22 [12, 24]	0.012
PICU mortality (%)	10 (32)	7 (24)	3 (8)	0.039

*ARDS* acute respiratory distress syndrome, *OI* oxygenation index, *PRISM III* Pediatric Risk of Mortality III, *VFDs* ventilator-free days

**Fig. 2:**
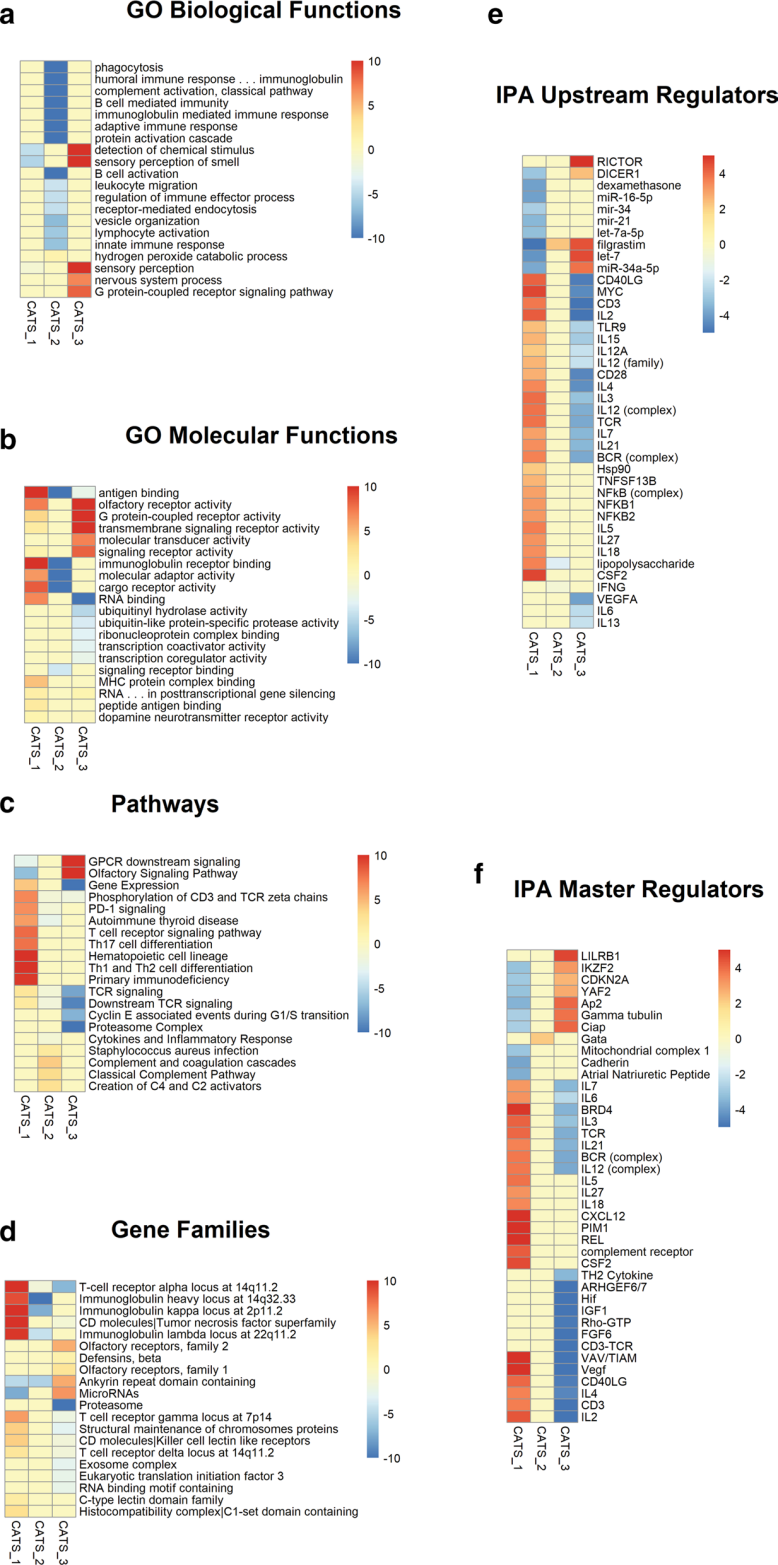
Heatmap of over- and under-expressed functional pathways and regulators using Gene Ontology (GO) and Ingenuity Pathway Analysis (IPA). The scale for A to D represents − log_10_(*q* value) for upregulated and log_10_(*q* value) for downregulated terms. Color scale represents activation/inhibition score for E and F

To understand the biology of the sub-phenotypes, we analyzed the association between sub-phenotype and total leukocytes, ANC, and ALC (Additional file 1: Table 1). All leukocyte metrics were associated with CATS subtypes, with modest overall effect sizes (η^2^) between 5.4 and 11.2%. We performed analyses assessing for upstream regulators, Gene Ontogeny terms, and key pathways (Fig. 2; Additional file 1: Figs. 2–7). CATS-1 was enriched for adaptive immune and T cell pathways. CATS-2 was enriched for complement pathways. CATS-3 showed upregulation of G-protein receptor signaling and olfactory pathways. Regulator analysis demonstrated significant inflammatory cytokine regulation of CATS-1 pathways.

**Fig. 3:**
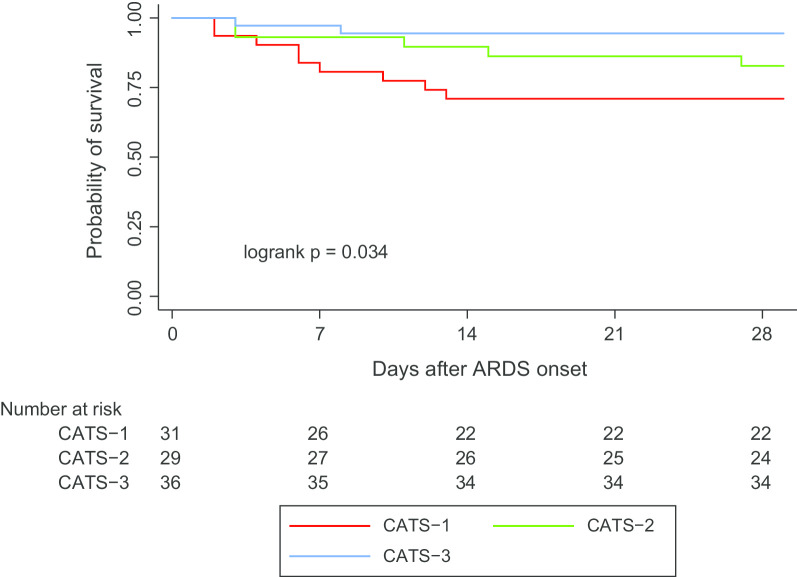
Kaplan–Meier survival curves for the CHOP ARDS Transcriptomic Subtypes (CATS); overall log-rank is significant (*p* = 0.034); in pairwise comparisons, the comparison between CATS-1 and CATS-3 reached statistical significance (*p* = 0.010)

In unadjusted analysis, CATS-3 had better survival and more VFDs than the other subtypes (Table 1, Fig. 3). After adjustment for PRISM III and immunocompromised status (Table 2), CATS-3 remained associated with lower mortality (OR 0.18, 95% CI 0.04–0.86) and higher probability of extubation (subdistribution HR 2.39, 95% CI 1.32–4.32). Adjustment for PRISM III strengthened this association, whereas adjustment for immunocompromised status attenuated it. Results were unchanged when also adjusting for ANC or ALC. We found similar results when we adjusted for the probability of death based on a published prediction model (Additional file 1: Table 2). The association of CATS-3 with better outcomes was not completely explained by fewer immunocompromised subjects in CATS-3, as an analysis restricted to immunocompetent subjects had point estimates confirming the association with lower mortality and greater VFDs in CATS-3 (Additional file 1: Table 3), although not all analyses reached statistical significance with the reduced sample size.

**Table 2:** Logistic regression and competing risk regression assessing association of CHOP ARDS Transcriptomic Subtypes (CATS) clusters and PICU mortality or probability of extubation by day 28 (accounting for the competing risk of death)

	PICU mortality		Probability of extubation	
	OR (95% CI)^a^	*p* value	SHR (95% CI)^b^	*p* value
Unadjusted				
CATS-1	Ref	–	Ref	–
CATS-2	0.67 (0.21–2.08)	0.487	1.01 (0.54–1.91)	0.967
CATS-3	0.19 (0.05–0.78)	0.021	2.15 (1.26–3.64)	0.005
Adjusted for PRISM III				
CATS-1	Ref	–	Ref	–
CATS-2	0.53 (0.16–1.76)	0.300	1.14 (0.60–2.17)	0.686
CATS-3	0.13 (0.03–0.60)	0.009	2.83 (1.55–5.17)	0.001
Adjusted for immunocompromised				
CATS-1	Ref	–	Ref	–
CATS-2	0.44 (0.12–1.58)	0.207	1.20 (0.66–2.18)	0.557
CATS-3	0.24 (0.06–1.07)	0.061	1.74 (1.02–3.00)	0.044
Adjusted for PRISM III + immunocompromised				
CATS-1	Ref	–	Ref	–
CATS-2	0.35 (0.09–1.36)	0.130	1.35 (0.74–2.44)	0.329
CATS-3	0.18 (0.04–0.86)	0.031	2.39 (1.32–4.32)	0.004
Adjusted for PRISM + immunocompromised + ANC				
CATS-1	Ref	–	Ref	–
CATS-2	0.28 (0.07–1.14)	0.074	1.44 (0.78–2.67)	0.247
CATS-3	0.11 (0.02–0.60)	0.011	2.77 (1.38–5.57)	0.004
Adjusted for PRISM + immunocompromised + ALC				
CATS-1	Ref	–	Ref	–
CATS-2	0.42 (0.10–1.67)	0.221	1.25 (0.69–2.27)	0.456
CATS-3	0.19 (0.04–0.90)	0.036	2.37 (1.32–4.25)	0.004

*ALC* absolute lymphocyte count, *ANC* absolute neutrophil count, *PRISM III* Pediatric Risk of Mortality III

^a^Odds ratio (OR) < 1: lower odds of mortality

^b^Subdistribution hazard ratio (SHR) > 1: greater hazard for extubation alive (i.e., shorter duration of ventilation)

## Discussion

We identified three sub-phenotypes of pediatric ARDS with distinct biologic pathways and prognoses using whole blood transcriptomics within 24 h of ARDS onset. The sub-phenotypes demonstrated some overlap of traditional clinical characteristics of ARDS severity, with immunocompromised status, stem cell transplant, and severe hypoxemia seen at differing proportions across all subtypes. Transcriptomic sub-phenotypes may provide insight into molecular mechanisms underlying pediatric ARDS heterogeneity, particularly when combined with clinical characteristics.

ARDS heterogeneity has contributed to the paucity of therapies, and subclassification into subtypes has been proposed as a way to address this. ARDS has been divided into direct or indirect [33–35], infectious or noninfectious [36, 37], focal versus nonfocal [38], and on the basis of biomarkers [11, 33]. A recent trial attempted predictive enrichment by stratifying treatment arm based on radiographic classification of focal or nonfocal ARDS [39]. A limitation of this approach in this trial was the imprecision of the clinical designation of focal versus nonfocal ARDS, with 21% of subjects misclassified. Thus, while clinical variables such as risk factors and comorbidities can inform heterogeneity, these terms remain imprecise.

Biomarker- and transcriptomic-based sub-phenotyping may offer some advantages, including greater insight into pathophysiology. Re-analysis of adult ARDS trials has identified hyper- and hypo-inflammatory sub-phenotypes characterized, in part, by differential levels of inflammatory biomarkers [11–13] and gene expression [40]. These findings in adults, and our results in pediatrics, demonstrate the utility of transcriptomics to uncover mechanisms underlying subtypes. Indeed, transcriptomics offer higher dimensional analysis, relative to protein biomarkers, a fact which potentially allows for better discrimination of sub-phenotypes.

We have previously demonstrated that infectious ARDS and noninfectious ARDS have different predictors of mortality [37]. CATS sub-phenotypes did not stratify according to either direct/indirect or infectious/noninfectious classifications. This may reflect the imprecision of clinical subtyping, different underlying biologies between clinical characterization and peripheral gene expression, or low power. However, clinical characteristics may potentially serve as one level of subclassification which can be improved upon with the addition of transcriptomics. Full realization of this requires more rapid turnaround for biologic-based sub-phenotyping, as clinical categorization is immediately applicable at bedside.

CATS sub-phenotypes revealed mechanisms which were not immediately apparent. CATS-1, for example, was enriched in adaptive immunity, which could be related to its relatively higher ALC. CATS-1 also demonstrated persistent hypoxemia, which is potentially related to signaling associated with adaptive immunity or to the types of organisms which may have caused the ARDS. CATS-2, which had nearly half of its subjects immunocompromised, was enriched in complement-related pathways, consistent with an emerging role for this pathway with stem cell transplant patients [41]. CATS-3 had suppression of adaptive immune and T cell receptor pathways. The sub-phenotypes also demonstrated prognostic utility, with CATS-3 subjects demonstrating improved survival and VFDs in unadjusted and adjusted analyses.

There are few trials in pediatric ARDS, and management is largely extrapolated from adults. The identification of sub-phenotypes with divergent biology forms the premise for targeted treatment. Subtypes with differential upregulation of innate and adaptive immunity offer intriguing opportunities for predictive enrichment in future trials of immunomodulatory therapies. Transcriptomics also allows insight into the mechanisms underlying the broader condition of ARDS, as well as the pathophysiology underlying different subtypes. ARDS has long been considered a disease of predominantly neutrophil infiltration [42, 43]. However, leukocyte populations and pathways other than innate immune hyperinflammation contribute to ARDS pathogenesis, which can potentially be dissected via transcriptomics [44, 45].

Given the ARDS heterogeneity, transcriptomic differences between the CATS sub-phenotypes may simply reflect differences in underlying risk factors, limiting their utility for predictive enrichment. However, the molecular basis for the heterogeneity of risk factors is also poorly elucidated. Pathway enrichment of the CATS sub-phenotypes provides insights into the different immune pathways implicated in early ARDS. Whether this can assist with predictive enrichment remains to be demonstrated. However, given the differences in mortality rate, these sub-phenotypes may also have a role for prognostic enrichment.

We performed microarray rather than direct RNA sequencing (RNA-seq). While RNA-seq provides greater dynamic range and is superior at identifying low-abundance transcripts, whole blood presents unique challenges. Up to 70% of the mRNA in a blood total RNA sample can be globin mRNA, with the remaining total RNA composed of > 90% ribosomal RNA (rRNA). Neither globin mRNA nor rRNA sequences contribute high-value information, and unlike hybridization techniques, overrepresentation of noninformative sequences consumes reagents and requires greater sequencing depth to yield useful information. Globin- and rRNA-depletion techniques are available [46, 47]; however, depletion techniques reduce the amount of RNA (particularly from leukopenic subjects) and potentially introduce artifact. Since microarrays are based on hybridization, over-abundance of globin or rRNA is less problematic, and so microarray was chosen for this study. Notably, every whole blood transcriptomic sub-phenotyping study to date has used microarray [15, 16, 48]. However, as RNA-seq technology improves and achieves better performance in whole blood, future transcriptomic studies may benefit from the improved coverage of direct sequencing technologies.

Our study has several strengths. We prospectively collected blood ≤ 24 h of ARDS onset and generated expression profiles in > 90% of samples. Detailed clinical data were collected and correlated with sub-phenotypes. However, our study has important limitations. Subjects were recruited from a single center, which may limit generalizability. However, demographics and severity of ARDS are comparable to other published cohorts [6, 49–51]. We did not use the recent Pediatric Acute Lung Injury Consensus Conference (PALICC) definition of pediatric ARDS [52], which allows unilateral infiltrates and has a specific SpO_2_-based severity stratification. Cohorts defined using PALICC may differ from ours in important ways which limit generalizability. Our sample size was small and only collected at ARDS onset, limiting our ability to fully characterize the subtypes, assess their temporal stability, and detect associations with outcomes. Our small sample size and low mortality rate precluded adjustment for multiple potential confounders. We sampled the blood, which while accessible, may not best reflect the transcriptome most relevant for ARDS. Alveolar sampling is uncommon in pediatrics and impractical for most clinical trial purposes. A future goal will be to reduce the number of transcripts required to discriminate between sub-phenotypes and operationalize a subtyping strategy. We did not include an external control population to assess up- or downregulation of pathways, relative to a non-ARDS cohort. Most importantly, our study lacks a validation cohort to assess the robustness of the CATS sub-phenotypes. This is the first transcriptomic study of pediatric ARDS, and validation cohorts with mRNA collection are lacking. Future studies of pediatric ARDS with transcriptomics are needed to assess for reproducibility of the CATS sub-phenotypes. Development of a reduced gene signature would simplify this process and is the focus of current work. Future cohorts should have parallel efforts correlating transcriptomics with plasma biomarkers, as a protein biomarker-based signature would likely prove faster, cheaper, and less labor-intensive. Biomarkers could also delineate mechanisms underlying the sub-phenotypes and facilitate comparisons with adult sub-phenotypes which have largely been defined using plasma proteins [11–13]. Re-analyses of adult ARDS trials have suggested differential treatment response based on subtype. To reproduce this in children, future trials in pediatric ARDS should collect both plasma for proteins and whole blood mRNA for transcriptomics and test treatment response by sub-phenotypes, as differences between adult and pediatric ARDS do not necessarily allow for translation of adult trial data to children.

## Conclusions

We identified three sub-phenotypes of pediatric ARDS using whole blood transcriptomics. The subtypes had differing clinical characteristics and divergent prognoses. Further studies should validate these findings and investigate mechanisms underlying differences between sub-phenotypes. Our results are the first steps toward reducing heterogeneity and designing trials of targeted, precision therapies in pediatric ARDS.

## Supplementary information

**Additional file 1.** Data Supplement.

## Data Availability

The clinical datasets used and/or analyzed during the current study are available from the corresponding author on reasonable request. Microarray data are available in the Gene Expression Omnibus (GSE147902).

## Abbreviations

ALC: Absolute lymphocyte count
ANC: Absolute neutrophil count
ARDS: Acute respiratory distress syndrome
CATS: CHOP ARDS Transcriptomic Subtypes
CHOP: Children’s Hospital of Philadelphia
DEGs: Differentially expressed genes
IPA: Ingenuity Pathway Analysis
OI: Oxygenation index
PEEP: Positive end-expiratory pressure
PICU: Pediatric intensive care unit
PRISM: Pediatric Risk of Mortality
RNA: Ribonucleic acid
VFDs: Ventilator-free days
