# Supplementary material for: Peripheral blood transcriptomic sub-phenotypes of pediatric acute respiratory distress syndrome
Source: Crit Care. 2020 Dec 7;24:681. doi: 10.1186/s13054-020-03410-7 (PMC7720038; doi:10.1186/s13054-020-03410-7)

**Peripheral blood transcriptomic sub-phenotypes of pediatric acute respiratory distress syndrome – DATA SUPPLEMENT**

Nadir Yehya, MD, MSCE^a^; Brian M. Varisco, MD^b,c^; Neal J. Thomas, MD^d^; Hector R. Wong, MD^b,c^; Jason D. Christie, MD, MSCE^e,f,g^; Rui Feng, PhD^h^

^a^ Department of Anesthesiology and Critical Care Medicine, Children’s Hospital of Philadelphia and University of Pennsylvania, Philadelphia, Pennsylvania, USA

^b^ Department of Pediatrics, Division of Critical Care Medicine, Cincinnati Children's Hospital Medical Center , Cincinnati, Ohio, USA

^c^ College of Medicine, University of Cincinnati , Cincinnati, Ohio, USA

^d^ Department of Pediatrics and Public Health Science, Division of Pediatric Critical Care Medicine, Penn State Hershey Children's Hospital, Hershey, Pennsylvania, USA

^e^ Department of Medicine, Pulmonary, Allergy, and Critical Care Division, Perelman School of Medicine, University of Pennsylvania, Philadelphia, Pennsylvania, USA

^f^ Center for Translational Lung Biology, Perelman School of Medicine, University of Pennsylvania, Philadelphia, Pennsylvania, USA

^g^ Center for Clinical Epidemiology and Biostatistics, Perelman School of Medicine, University of Pennsylvania, Philadelphia, Pennsylvania, USA

^h^ Department of Biostatistics, Center for Clinical Epidemiology and Biostatistics, Epidemiology, and Informatics, University of Pennsylvania, Philadelphia, Pennsylvania, USA

**Corresponding author:**

Nadir Yehya. MD, MSCE

Department of Anesthesiology and Critical Care Medicine

6040A Wood Building, Children’s Hospital of Philadelphia

3401 Civic Center Boulevard

Philadelphia, Pennsylvania 19104

USA

[yehyan@email.chop.edu](mailto:yehyan@email.chop.edu)

**Supplementary Table 1:** Leukocytes stratified by CHOP ARDS Transcriptomic Subtypes (CATS)

| **Variables** | **CATS-1**  **(n = 31)** | **CATS-2**  **(n = 29)** | **CATS-3**  **(n = 36)** | ***p* value** | **η^2^** |
| --- | --- | --- | --- | --- | --- |
| White blood cells (K/µL) | 10 [5.2, 12.5] | 9.7 [3.5, 15] | 15.8 [12, 20.1] | 0.001 | 0.075 |
| Subsets  ANC (K/µL)  ANC (percentage)  ALC (K/µL)  ALC (percentage) | 4.9 [2.9, 9.5]  70 [53, 88]  1.6 [0.7, 3.3]  16 [8, 34] | 7.3 [2.7, 11.8]  79 [67, 89]  0.5 [0.3, 1.1]  6 [3, 15] | 12.8 [7.4, 16.2]  82 [72, 89]  1.1 [0.7, 2.9]  9 [5, 17] | < 0.001  0.027  < 0.001  0.001 | 0.112  0.054  0.061  0.093 |

**Supplementary Table 2:** Logistic regression and competing risk regression assessing association of CHOP ARDS Transcriptomic Subtypes (CATS) clusters and PICU mortality or probability of extubation by day 28 (accounting for the competing risk of death) when adjusted for predicted mortality (mortality prediction model = OI at 6 hours, PELOD2 organ failure score, vasopressor score, and immunocompromised status)

|  | **PICU mortality** | | **Probability of extubation** | |
| --- | --- | --- | --- | --- |
|  | **OR (95% CI)^a^** | **p value** | **SHR (95% CI)^b^** | **p value** |
| **Unadjusted**  **CATS-1**  **CATS-2**  **CATS-3** | Ref  0.67 (0.21 to 2.08)  0.19 (0.05 to 0.78) | -  0.487  0.021 | Ref  1.01 (0.54 to 1.91)  2.15 (1.26 to 3.64) | -  0.967  0.005 |
| **Adjusted for mortality risk model**  **CATS-1**  **CATS-2**  **CATS-3** | Ref  0.71 (0.16 to 3.14)  0.18 (0.03 to 0.99) | -  0.635  0.049 | Ref  0.87 (0.47 to 1.61)  1.78 (1.06 to 2.97) | -  0.659  0.029 |
| **Adjusted for mortality risk model + ANC**  **CATS-1**  **CATS-2**  **CATS-3** | Ref  0.70 (0.16 to 3.09)  0.14 (0.02 to 0.90) | -  0.638  0.038 | Ref  0.88 (0.47 to 1.62)  1.85 (1.06 to 3.23) | -  0.676  0.031 |
| **Adjusted for mortality risk model + ALC**  **CATS-1**  **CATS-2**  **CATS-3** | Ref  0.70 (0.15 to 3.26)  0.18 (0.03 to 0.99) | -  0.647  0.049 | Ref  0.87 (0.47 to 1.63)  1.77 (1.06 to 2.97) | -  0.666  0.029 |

ALC: absolute lymphocyte count; ANC: absolute neutrophil count; PRISM III: Pediatric Risk of Mortality III

^a^ Odds ratio (OR) < 1: lower odds of mortality

^b^ Subdistribution hazard ratio (SHR) > 1: greater hazard for extubation alive (i.e., shorter duration of ventilation)

**Supplementary Table 3:** Logistic regression and competing risk regression assessing association of CHOP ARDS Transcriptomic Subtypes (CATS) clusters and PICU mortality or probability of extubation by day 28 (accounting for the competing risk of death) when restricted to immunocompetent subjects (n = 67, 7 deaths)

|  | **PICU mortality** | | **Probability of extubation** | |
| --- | --- | --- | --- | --- |
|  | **OR (95% CI)^a^** | **p value** | **SHR (95% CI)^b^** | **p value** |
| **Unadjusted**  **CATS-1 (n = 21, 4 deaths)**  **CATS-2 (n = 15, 1 death)**  **CATS-3 (n = 31, 2 deaths)** | Ref  0.30 (0.03 to 3.04)  0.29 (0.05 to 1.77) | -  0.310  0.181 | Ref  1.25 (0.62 to 2.50)  1.56 (0.90 to 2.80) | -  0.530  0.136 |
| **Adjusted for PRISM III**  **CATS-1**  **CATS-2**  **CATS-3** | Ref  0.20 (0.01 to 2.59)  0.14 (0.02 to 1.14) | -  0.216  0.066 | Ref  1.47 (0.73 to 2.95)  2.44 (1.24 to 4.81) | -  0.282  0.010 |
| **Adjusted for PRISM + ANC**  **CATS-1**  **CATS-2**  **CATS-3** | Ref  0.14 (0.01 to 1.90)  0.06 (0.01 to 0.74) | -  0.139  0.026 | Ref  1.51 (0.74 to 3.08)  2.63 (1.29 to 5.64) | -  0.253  0.013 |
| **Adjusted for PRISM + ALC**  **CATS-1**  **CATS-2**  **CATS-3** | Ref  0.25 (0.19 to 3.35)  0.11 (0.01 to 1.09) | -  0.296  0.059 | Ref  1.35 (0.67 to 2.74)  2.46 (1.27 to 4.76) | -  0.400  0.008 |

ALC: absolute lymphocyte count; ANC: absolute neutrophil count; PRISM III: Pediatric Risk of Mortality III

^a^ Odds ratio (OR) < 1: lower odds of mortality

^b^ Subdistribution hazard ratio (SHR) > 1: greater hazard for extubation alive (i.e., shorter duration of ventilation)

**Supplementary Figure 1:** Gap statistic and 95% confidence intervals for *k* = 1 through 10.


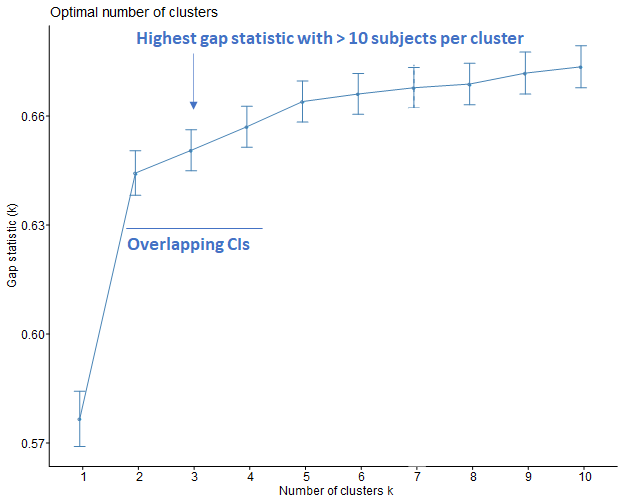


**Supplementary Figure 2:** Heatmap of over- and under-expressed (q < 0.1) Gene Ontology (GO) Biological Processes. The scale -log_10_(q value) for up- and log_10_(q value) for downregulated terms.


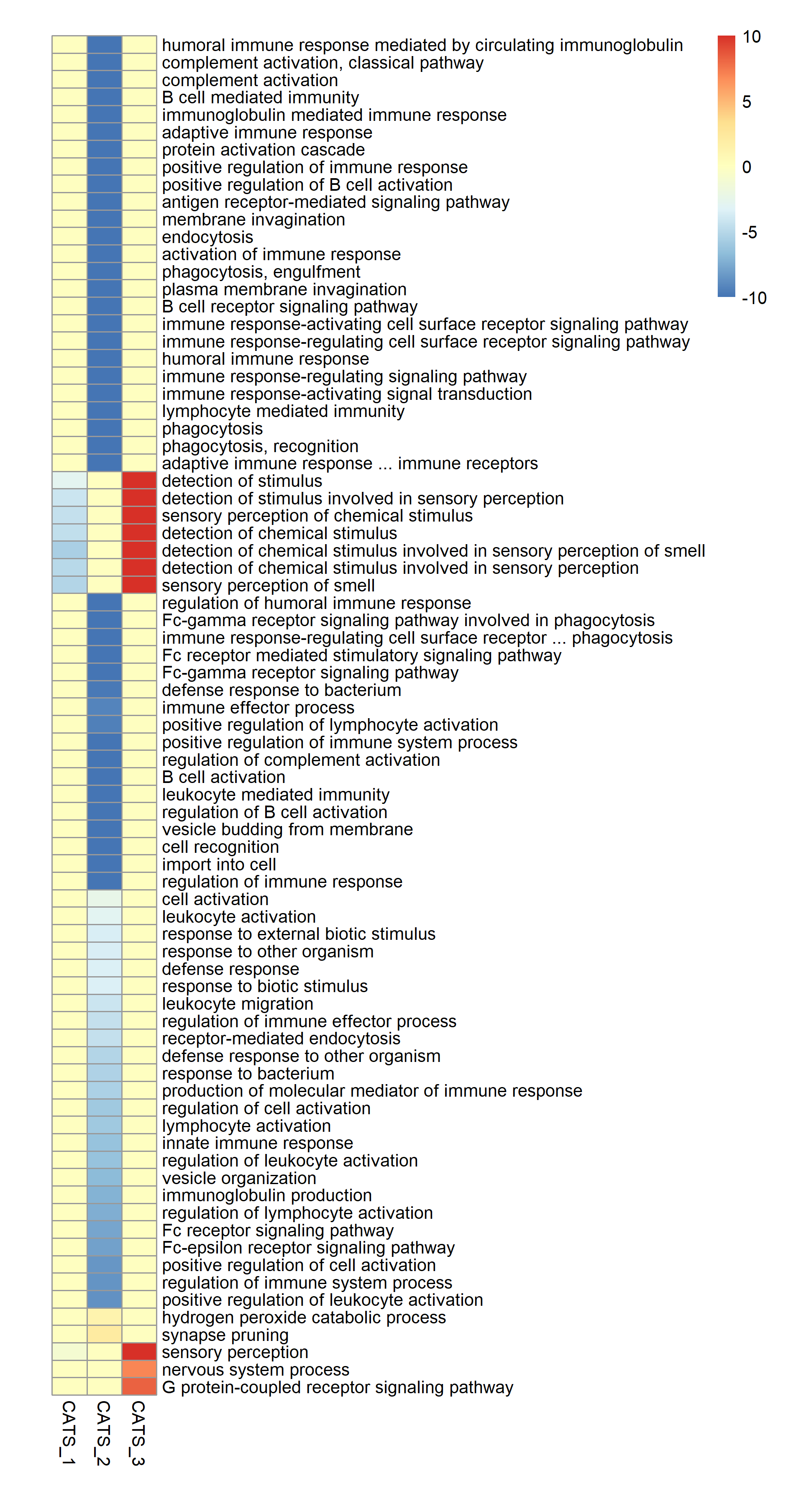


**Supplementary Figure 3:** Heatmap of over- and under-expressed (q < 0.1) Gene Ontology (GO) Molecular Functions. The scale -log_10_(q value) for up- and log_10_(q value) for downregulated terms.


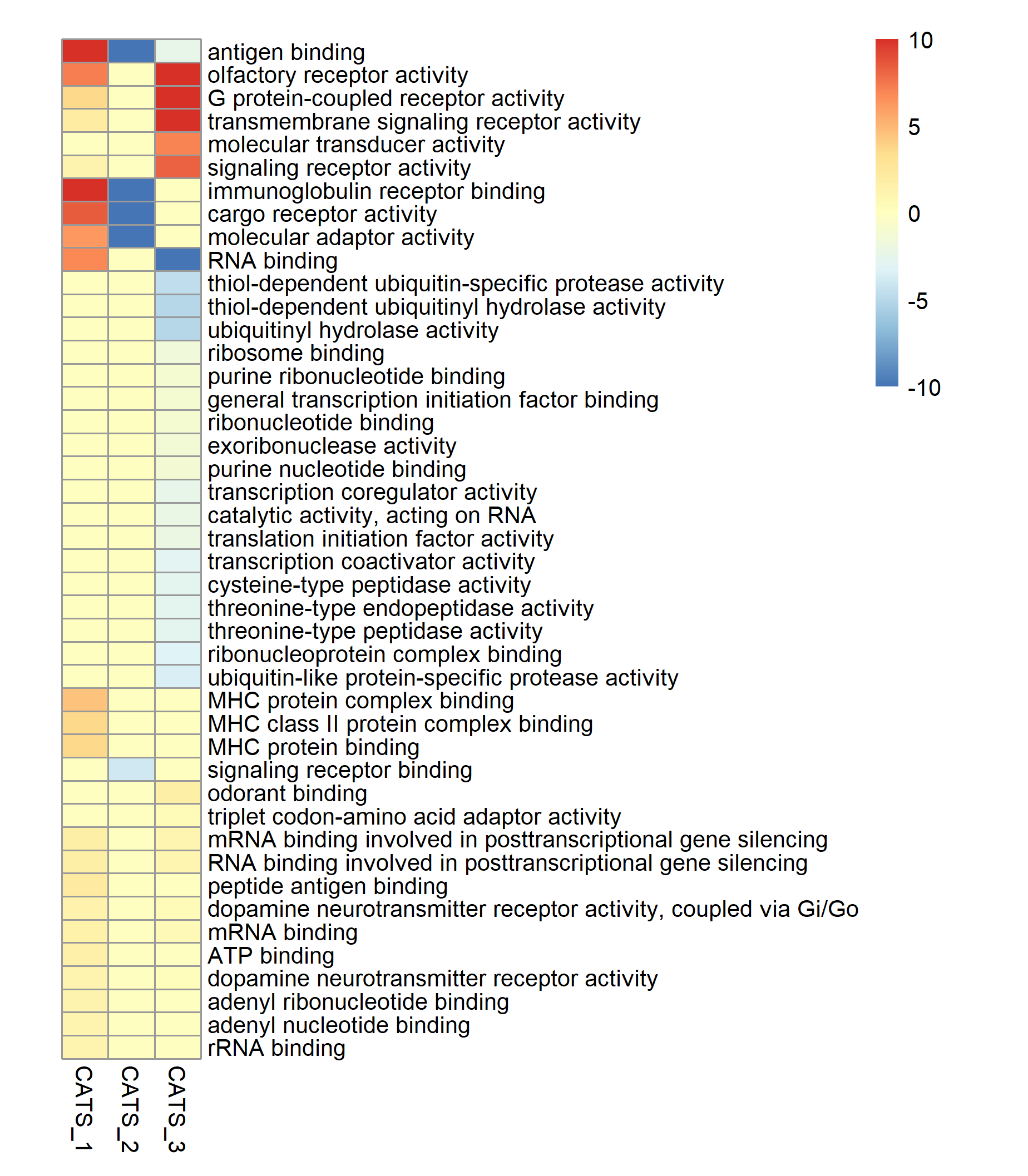


**Supplementary Figure 4:** Heatmap of over- and under-expressed (q < 0.1) Ingenuity Pathway Analysis (IPA) Pathways. The scale -log_10_(q value) for up- and log_10_(q value) for downregulated terms.


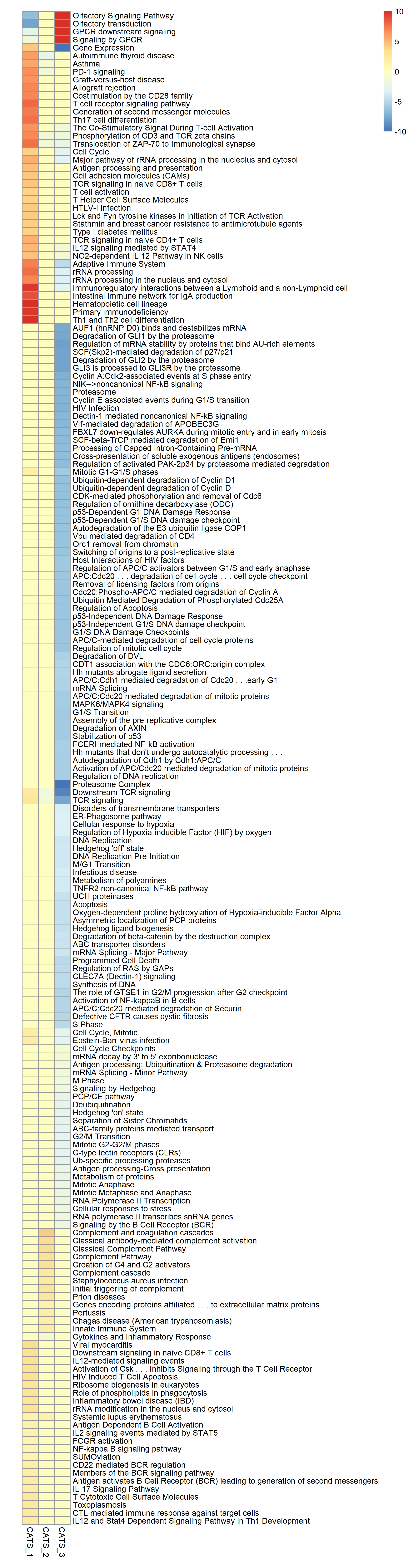


**Supplementary Figure 5:** Heatmap of over- and under-expressed (q < 0.1) Ingenuity Pathway Analysis (IPA) Gene Families. The scale -log_10_(q value) for up- and log_10_(q value) for downregulated terms.


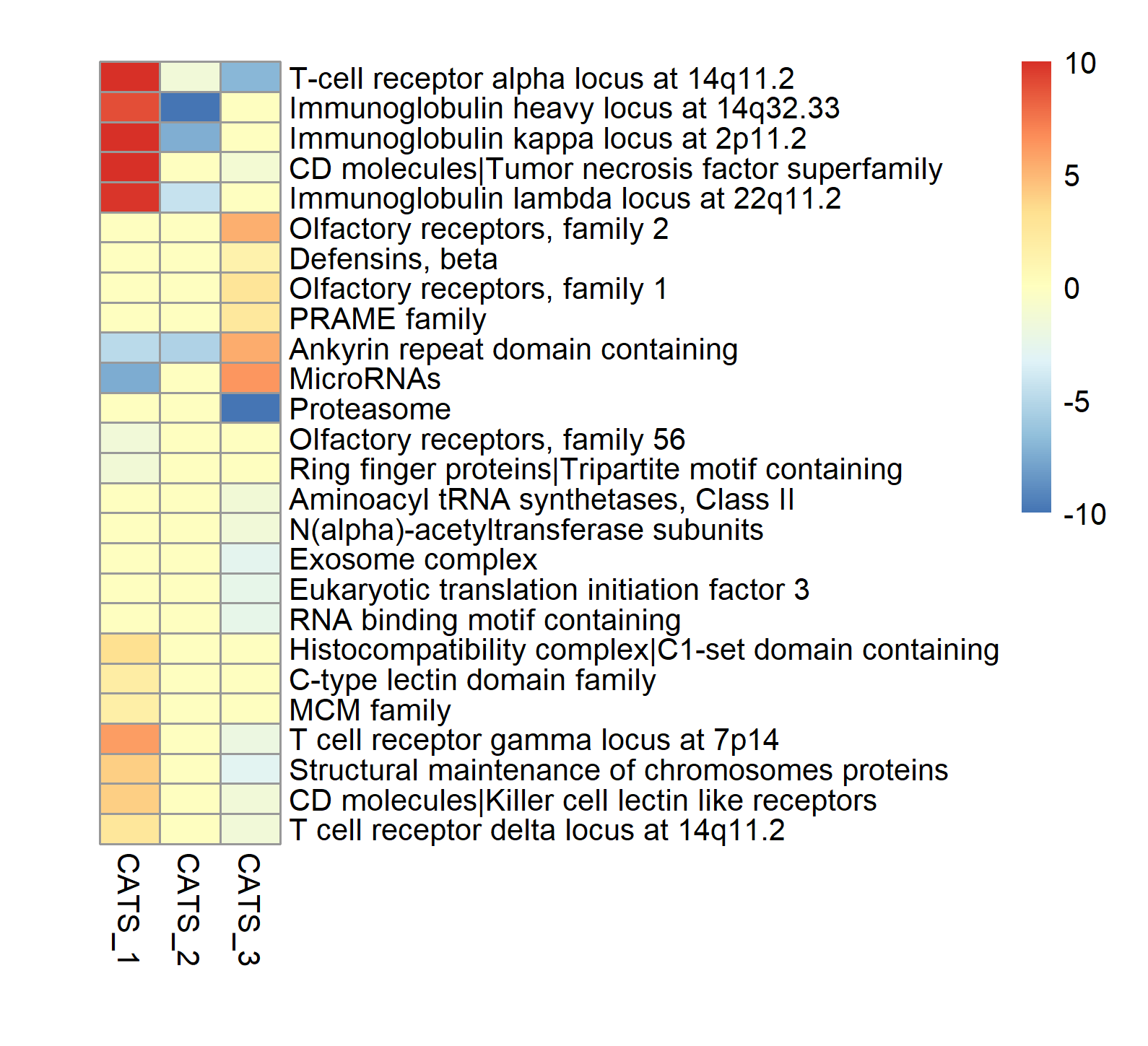


**Supplementary Figure 6:** Heatmap of activated and inhibited (q < 0.1) Ingenuity Pathway Analysis (IPA) Upstream Regulators. The scale -log_10_(q value) for activators and log_10_(q value) for inhibited regulator terms.


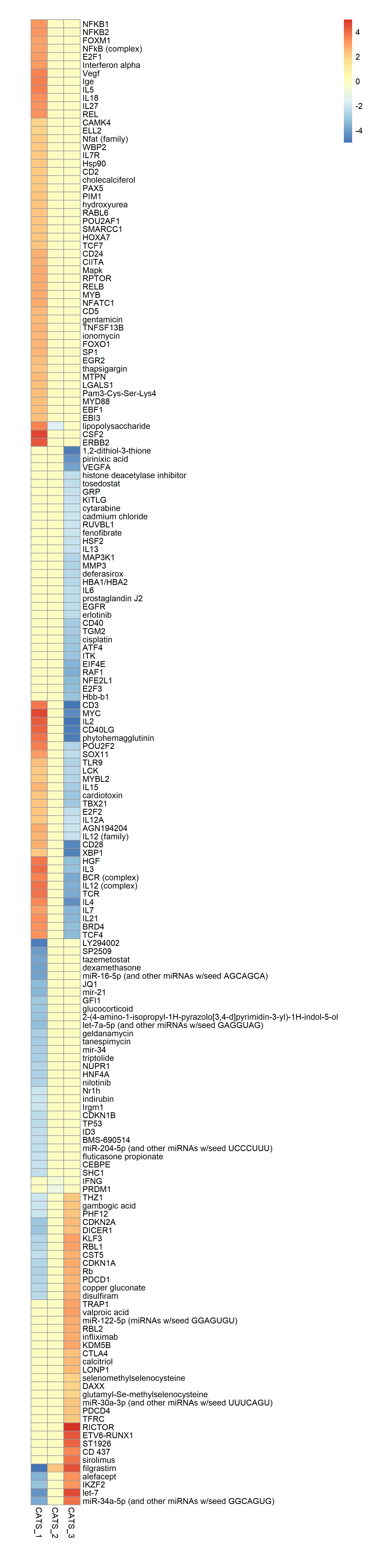


**Supplementary Figure 7:** Heatmap of activated and inhibited (q < 0.1) Ingenuity Pathway Analysis (IPA) Master Regulators. The scale -log_10_(q value) for activators and log_10_(q value) for inhibited regulator terms.


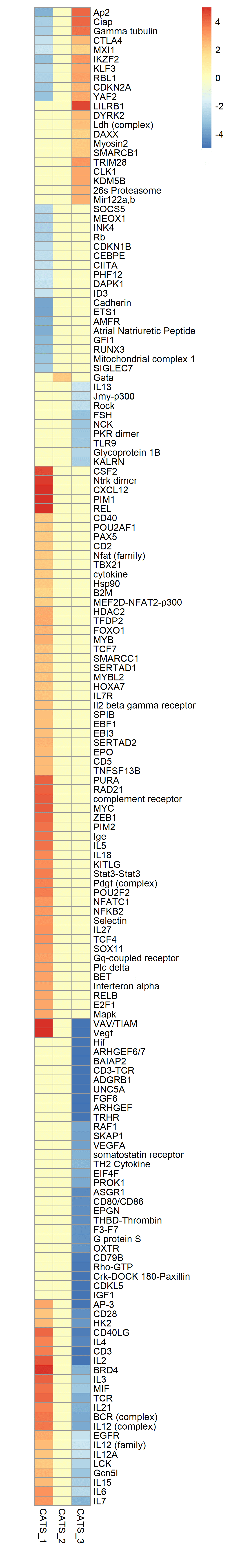

Supplement: Supplementary file 1 — Additional file 1. Data Supplement. [file 13054_2020_3410_MOESM1_ESM.docx]
